# Dietary Annatto-Extracted Tocotrienol Reduces Inflammation and Oxidative Stress, and Improves Macronutrient Metabolism in Obese Mice: A Metabolic Profiling Study

**DOI:** 10.3390/nu13041267

**Published:** 2021-04-13

**Authors:** Chwan-Li Shen, Sivapriya Ramamoorthy, Gurvinder Kaur, Jannette M. Dufour, Rui Wang, Huanbiao Mo, Bruce A. Watkins

**Affiliations:** 1 Department of Pathology, Texas Tech University Health Sciences Center, Lubbock, TX 79430, USA; rui.wang@ttuhsc.edu; 2 Center of Excellence for Integrative Health, Texas Tech University Health Sciences Center, Lubbock, TX 79430, USA; Gurvinder.Kaur@ttuhsc.edu (G.K.); Jannette.Dufour@ttuhsc.edu (J.M.D.); 3 Center of Excellence for Translational Neuroscience and Therapeutics, Texas Tech University Health Sciences Center, Lubbock, TX 79430, USA; 4 Metabolon, Inc., Morrisville, NC 27560, USA; SRamamoorthy@metabolon.com; 5 Department of Medical Education, Texas Tech University Health Sciences Center, Lubbock, TX 79430, USA; 6 Department of Cell Biology and Biochemistry, Texas Tech University Health Sciences Center, Lubbock, TX 79430, USA; 7 Department of Nutrition, Georgia State University, Atlanta, GA 30303, USA; hmo@gsu.edu; 8 Department of Nutrition, University of California at Davis, Davis, CA 95616, USA

**Keywords:** vitamin E, tocotrienol, metabolites, obesity, mice, inflammation

## Abstract

Obesity and its related complications are a world-wide health problem. Dietary tocotrienols (TT) have been shown to improve obesity-associated metabolic disorders, such as hypercholesterolemia, hyperglycemia, and gut dysbiosis. This study examined the hypothesis that the antioxidant capacity of TT alters metabolites of oxidative stress and improves systemic metabolism. C57BL/6J mice were fed either a high-fat diet (HFD control) or HFD supplemented with 800 mg annatto-extracted TT/kg (HFD+TT800) for 14 weeks. Sera from obese mice were examined by non-targeted metabolite analysis using UHPLC/MS. Compared to the HFD group, the HFD+TT800 group had higher levels of serum metabolites, essential amino acids (lysine and methionine), sphingomyelins, phosphatidylcholine, lysophospholipids, and vitamins (pantothenate, pyridoxamine, pyridoxal, and retinol). TT-treated mice had lowered levels of serum metabolites, dicarboxylic fatty acids, and inflammatory/oxidative stress markers (trimethylamine N-oxide, kynurenate, 12,13-DiHOME, and 13-HODE + 9-HODE) compared to the control. The results suggest that TT supplementation lowered inflammation and oxidative stress (oxidized glutathione and GSH/GSSH) and improved macronutrient metabolism (carbohydrates) in obese mice. Thus, TT actions on metabolites were beneficial in reducing obesity-associated hypercholesterolemia/hyperglycemia. The effects of a non-toxic dose of TT in mice support the potential for clinical applications in obesity and metabolic disease.

## 1. Introduction

Obesity is characterized by an excessive amount of adipose tissue, which has been recognized as a major endocrine organ that releases a wide variety of signaling molecules (hormones, growth factors, cytokines and chemokines) [1,2]. These signaling molecules play central roles in the regulation of energy metabolism and homeostasis, inflammation, and immunity [1,2].

Obesity is accompanied by a state of chronic low-grade systemic inflammation that accelerates the development of insulin resistance in type 2 diabetes (T2DM) [3] and hyperlipidemia in fatty liver diseases and cardiovascular diseases [4]. Hyperglycemia and high intracellular glucose metabolism, a hallmark of T2DM, is associated with excessive mitochondrial reactive oxygen species (ROS) and oxidative stress [5], which can impair insulin signaling and lead to insulin resistance of skeletal muscle glucose transport [6]. Moreover, there is growing evidence that excess generation of ROS, largely due to hyperglycemia, causes oxidative stress in a variety of tissues [5].

Studies in cell cultures, animal models, and human participants have provided compelling evidence that vitamin E, mainly tocopherols, play a role in preventing T2DM due to their antioxidant and anti-inflammatory capacities [7]. Until recently, tocotrienols (TTs), vitamin E molecules that differ from tocopherols by possessing an unsaturated isoprenoid side chain, have been overlooked for their potential effects in preventing T2DM [8,9]. TTs are more lipophilic, making them more easily incorporated into the cell membrane [10] and more rapidly absorbed than tocopherols [11]. Among all eight vitamin E isomers, δ-TT is considered as the most potent antioxidant/anti-inflammatory agent [12]. δ-TT has also been shown to be more protective than α-tocopherol against chronic diseases, such as T2DM, obesity, metabolic syndrome, fatty liver diseases, and cardiovascular diseases [13].

Tocotrienols have received increased attention due to their ability to attenuate obesity-associated complications via various molecular/cellular mechanisms, such as induction of apoptosis in preadipocytes, inhibition of fat cell adipogenesis and differentiation, activation of energy sensing pathways, protection of cell membranes against oxidative damage, inhibition of lipid peroxidation, and suppression of inflammation [8,14]. In animal models of obesity and T2DM [8,9,15], the anti-proliferative, antioxidant and anti-inflammatory properties of TTs contributed to their effects on hypo-cholesterolemia [16] and improved glucose homeostasis [8,9]. TT supplementation improved lipid metabolism as shown in reduced adipocyte hypertrophy, serum free fatty acids, triglycerides, and cholesterol [8,17] and hepatic steatosis in obese mice with increased markers of fatty acid β-oxidation (i.e., carnitine palmitoyltransferase 1A, carnitine palmitoyltransferase 2, and Forkhead box A2) and reduced markers of fatty acid synthesis (e.g., fatty acid synthase, acetyl-co-carboxylase-1) in adipose tissue [8]. In addition, TT supplementation significantly improved glucose tolerance in obese mice, as shown by a decreased area under the curve of the glucose tolerance test [8,9], which in part is attributed to the suppression of tumor necrosis factor-α (TNF-α), interleukin-1 beta (IL-1β), and interleukin-6 (IL-6) in lipopolysaccharide-stimulated peritoneal macrophages of obese mice [18]. In humans, TT administration significantly reduced serum levels of total cholesterol, low density lipoproteins (LDL), and triglycerides [13].

The present study used global non-targeted metabolomics to determine the metabolic effects of TT supplementation in mice with high-fat-diet (HFD)-induced obesity. Our approach focused on six broad pathways involving the metabolism of lipids, amino acids, cofactors and vitamins, nucleotides, peptides, and xenobiotics. We hypothesized that a diet supplemented with TT would reduce lipid peroxidation and oxidative stress, and alter the metabolism of amino acids, fatty acids, phospholipids, and sphingolipids in obese mice in ways that would be beneficial on lipid metabolism and glucose homeostasis in obese mice.

## 2. Materials and Methods

### 2.1. Animals and Treatments

Twenty male C57BL/6J mice (5-week-old, Jackson Laboratory, Bar Harbor, ME, USA), housed in cages of four mice and maintained at a controlled temperature of 21 ± 2 °C with a 12 h light-dark cycle, were fed chow diet and distilled water at libitum for 5 days. After acclimation, mice were weighed and assigned to two groups: control group fed high-fat diet (HFD; 21%, 20%, and 58% of energy from protein, carbohydrates, and fat, respectively, Research Diets Inc., New Brunswick, NJ, USA) and the treatment group fed the HFD supplemented with annatto-extracted TT at 800 mg/kg diet (HFD+TT800). TT was extracted from annatto oil with 71.3% purity, containing 86.1% δ-TT and 13.9% γ-TT, free of tocopherol (American River Nutrition, LLC, Hadley, MA, USA). The remaining 30% components of TT are mainly fatty acids of annatto oil itself, with a very small amount of plant terpenes, sterols, and waxes.

TT was premixed with tocopherol-stripped soybean oil (Dytes Inc., Bethlehem, PA, USA) before being incorporated into the ingredients of the HFD. Both groups were given the diets for 14 weeks with free access to water and diet during the study period. Previous rodent studies showed the anti-inflammatory actions of δ-TT at 400 and 1600 mg/kg diet/ in obese mice [8]; the TT dose used in our current study, 800 mg/kg diet, is within this range. Table 1 lists the ingredient composition in g/kg of the two dietary groups. Body weight, food intake, and water consumption were recorded weekly. All conditions and handling of the mice were approved by the Texas Tech University Health Sciences Center Institutional Animal Care and Use Committee. All procedures and animal use were performed in accordance with the relevant guidelines and regulations.

### 2.2. Sample Collection

At the end of the experiment, mice were fasted for 4-h, anesthetized with isoflurane, and then euthanized. Blood was collected into microtainer blood collector tubes (BD Biosciences, San Jose, CA, USA) and then centrifuged at 1500× *g* for 20 min to isolate serum. The serum samples were kept at −80 °C until further analyses.

### 2.3. Metabolomics Analyses of Serum

Sample preparation and non-targeted metabolomics analysis of serum samples were conducted at Metabolon, Inc. (Morrisville, NC, USA) as described in a previous study [19]. Briefly, individual samples were subjected to methanol extraction and then split into aliquots for analysis by ultrahigh performance liquid chromatography/mass spectrometry (UHPLC/MS). The global biochemical profiling analysis comprised of four unique arms consisting of reverse phase chromatography positive ionization methods optimized for hydrophilic (LC/MS Pos Polar) and hydrophobic compounds (LC/MS Pos Lipid), reverse phase chromatography with negative ionization conditions (LC/MS Neg), and a HILIC chromatography method coupled to negative ionization (LC/MS Polar) [20]. All of the methods alternated between full scan MS and data dependent multi-stage mass spectrometry (MSn) scans. The scan range varied slightly between methods but generally covered 70–1000 *m*/*z*.

Metabolites were identified by automated comparison of the ion features in the experimental samples to a reference library of standard chemical entries [21] that included retention time, molecular weight (*m*/*z*), preferred adducts, in-source fragments, and associated MS spectra and curated by visual inspection for quality control using software developed at Metabolon, Inc. (Morrisville, NC, USA).

### 2.4. Statistical Analyses

Principal components analysis (PCA) and hierarchical clustering were used for classification analysis. For the scaled intensity graphics, each biochemical in original scale (raw area count) was rescaled to set the median across all animals and time-points as equal to 1. To gain insight into individual metabolites that may be able to differentiate the groups, we also performed random forest analysis. This method attempts to bin individual samples in groups based on their metabolite similarities and differences. The method is unbiased since the prediction for each sample is based on trees built from a subset of samples that do not include that sample [22]. Random forest also defines which metabolites contribute most strongly to the group binning.

Two types of statistical analyses for metabolites were performed: (1) significance tests; and (2) classification analysis. Standard statistical analyses were performed in ArrayStudio on log-transformed data. For analyses not standard in ArrayStudio, the R program (http://cran.r-project.org/ (accessed on 27 October 2016)) was used. Following log transformation and imputation of missing values, if any, with the minimum observed value for each compound, Welch’s two sample *t*-Test was used as significance test to identify biochemicals that differed significantly (*p <* 0.05) between experimental groups. An estimate of the false discovery rate (q-value) was calculated to take into account the multiple comparisons that normally occur in metabolomic-based studies.

## 3. Results

There were no differences in body weight, food intake, and water consumption between the HFD group and the HFD+TT800 group throughout the study period (data not shown).

### 3.1. Metabolite Summary and Significantly Altered Biochemicals

A comprehensive non-targeted mass spectrometry-based metabolomics profiling analysis was performed on sera from the HFD and HFD+TT800 mice after a 14-week feeding period. Overall, a total of 566 metabolites, or biochemicals, of known identity were detected and categorized into six broad categories including amino acids, cofactors and vitamins, lipids, nucleotides, peptides, and xenobiotics. Among these 566 compounds, 68 biochemicals were primarily responsible for the observed significant difference due to TT (*p ≤* 0.05) with 44 additional biochemicals showing a tendency of change due to TT (0.05 < *p <* 0.1). Among the 68 biochemicals (*p ≤* 0.05) with changes in concentration, 32 biochemicals edged higher whereas 36 others went lower. Similarly, among the 44 biochemicals (0.05 < *p <* 0.1), 18 biochemicals were higher whereas 26 others were lower.

**Figure 1:**
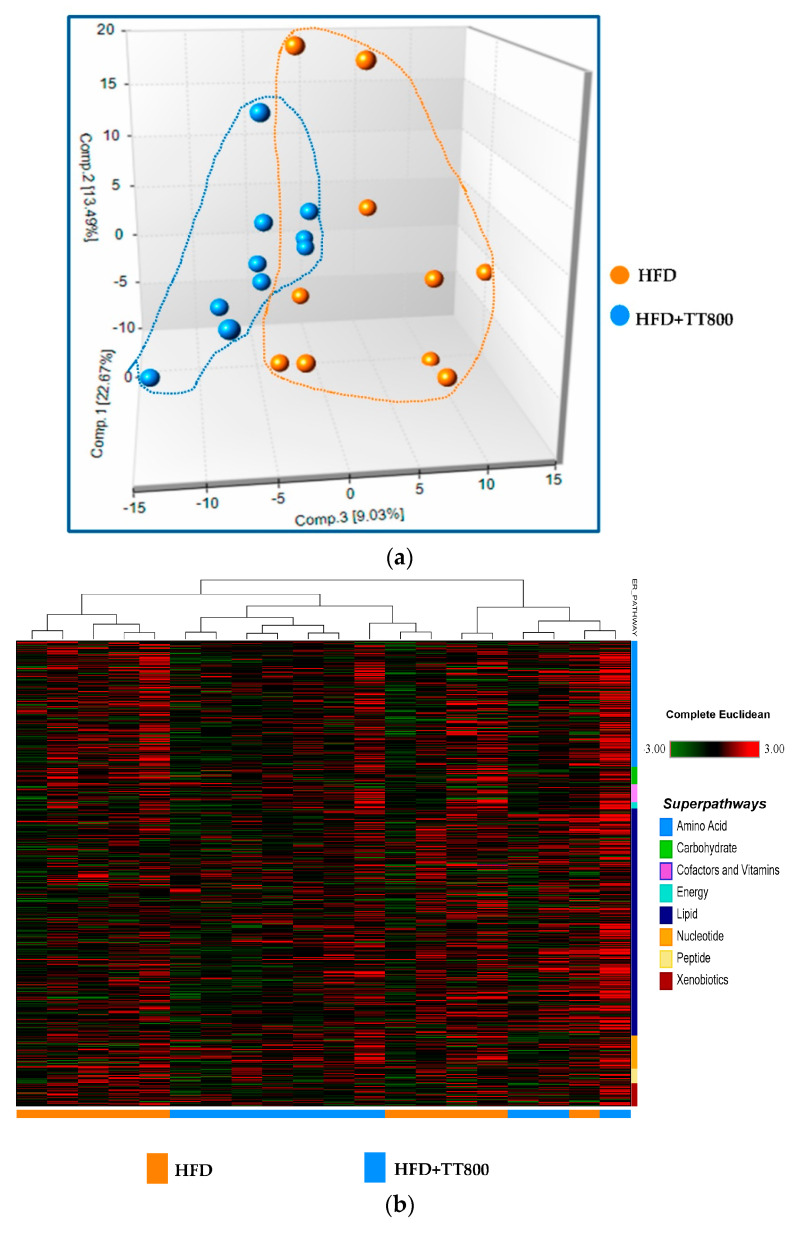

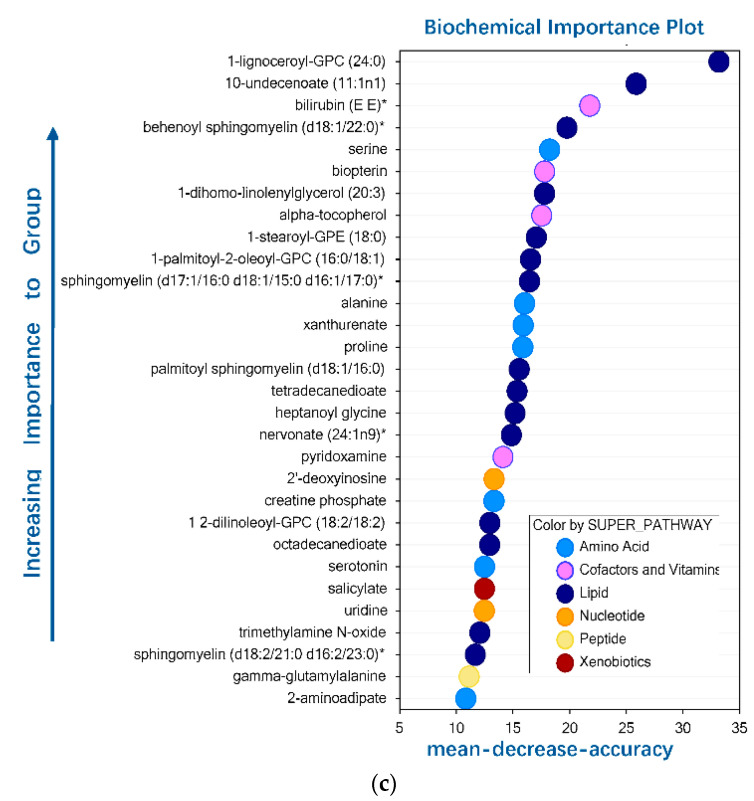
(**a**) Principal component analysis (PCA) showed differences in metabolites of serum samples in mice between the control HFD group and the treatment HFD+TT800 group. Each ball represents the cumulative metabolites from each mouse. The 10 orange balls are mice from the control HFD group and 10 blue balls are mice from the HFD+TT800 group. Metabolites associated with mice in the TT800 group are more tightly associated or closer together than those from mice in the control HFD group. TT supplementation appears to narrow the range of metabolite data in the mice fed the HFD. Each dietary group was comprised of *n* = 10 mice. (**b**) Differences in eight superpathways of serum metabolites between the control HFD group and the HFD+TT800 group. Heatmap of the hierarchical cluster analysis of serum metabolites by Student’s *t*-test to distinguish the eight superpathways of metabolites between the control HFD group and the HFD+TT800 group. The eight superpathways include amino acids, carbohydrates, cofactors and vitamins, energy metabolites, lipids, nucleotides, peptides, and xenobiotics. Color in red indicates up-regulation and color in green indicates down-regulation (fold changes, *p <* 0.05). Each dietary group was comprised of *n* = 10 mice. (**c**) Biochemical importance plot based on random forest classification of the overall metabolomics profile for mouse serum samples. Random forest analysis distinguishes between the control HFD group subsets of superpathways and the treated HFD+TT800 group subsets of superpathways. Progression to TT800 supplementation was set as the response variable and all serum metabolites or biochemicals identified by the platform were set as predictors. The biochemicals are plotted according to the increasing importance to group separation to elucidate the metabolic fingerprint for TT800 supplementation as compared to the control HFD group. The figure presents the 30 top-ranked metabolites and their classification (indicated in the figure, lower right) based on their importance for the identification of the two treatment subsets. Light blue = amino acid, pink = cofactors and vitamins, dark blue = lipid, orange = nucleotide, light yellow = peptide, burgundy = xenobiotics. Each dietary group was comprised of *n* = 10 mice.

The PCA analysis revealed adequate segregation of the HFD group from the HFD+TT800 group, suggesting distinct biochemical compositions (Figure 1a). About 30% of the samples from each group showed overlapping distribution, demonstrating similar biochemical composition between these groups. The observed variability in 30% of the samples from each group could be attributed to potential variances in sample collection that lead to global changes in the amount of metabolites in the samples.

Figure 1b presents a heatmap of hierarchical clustering of serum metabolites between the HFD and HFD+TT800 groups. Most of the HFD+TT800 biochemicals show a single branch of the dendrogram whereas the HFD samples segregate to a separate branch. The groups have eight distinguishable metabolic signatures/superpathways, including amino acids, carbohydrates, cofactors and vitamins, energy metabolites, lipids, nucleotides, peptides, and xenobiotics.

Figure 1c shows the biochemical importance plot based on the results of the random forest test. The 30 top ranking biochemical differences of importance to group classification separations are shown, indicating key differences in lipid metabolism and amino acid metabolism between the groups.

### 3.2. Effects of TT on Lipid Metabolism

The effects of TT supplementation on the serum level of fatty acid metabolites, presented as the ratio of (HFD+TT800)/HFD, are presented in Table 2. Relative to the HFD group, the HFD+TT800 group had lower serum levels of 13-methylmyristate (a branched chain fatty acid), two medium-chain fatty acids-pelargonate (C9:0) and 10-undecenoate (C11:1n1), and several fatty acid dicarboxylates, including azelate, sebacate, 1,11-undecanedicarboxylate, dodecanedioate, tetradecanedioate and octadecanedioate (*p ≤* 0.05). Other fatty acid dicarboxylates, including 2-hydroxyadipate, suberate, undecanedioate and hexadecanedioate, showed a trend of reduction (0.05 < *p <* 0.1). The HFD+TT800 group had lower levels of several fatty acid acyl glycine metabolites, including valerylglycine, hexanoylglycine, heptanoyl glycine, 3,4-methylene heptanoylglycine, and N-octanoylglycine, than the HFD group (*p ≤* 0.05). In addition, the HFD+TT800 group had altered fatty acid acyl carnitine metabolites—lower hexanoylcarnitine (C6) levels and higher lignoceroylcarnitine (C24) levels—and a higher level of serum carnitine (*p ≤* 0.05) (Table 3).

Some oxylipins (bioactive lipids) were changed favorably in mice supplemented with TT, as shown by the (HFD+TT800)/HFD ratio (Table 2). In this case, 9-HODE and 13-HODE and 14 HDoHE/17-HDoHE were lower, suggesting lower acute pain and inflammation and auto-oxidation of DHA. Thromboxane B2, an inactive metabolite of thromboxane A2, was lower as well for the ratio values (ratio 0.45). Furthermore, 14 HDoHe/17-HDoHe showed a lower ratio (ratio 0.72), and this lipokine appears to lower circulating triglycerides. 12-HETE, which activates protein kinase C and mediates biological functions of growth factor cytokines was lower. Interestingly, the ratio for 12 HHTrE, a COX metabolite of arachidonate that may promote or limit inflammatory and promote allergic responses, was lower with TT supplementation. In contrast, the ratios for oleoyl ethanolamide, palmitoyl ethanolamide and linoleoyl ethanolamide in serum were not different between the groups.

The effects of TT supplementation on sphingolipids, phospholipids, and other lipid metabolites are shown in Table 3. The HFD+TT800 group showed significantly higher concentrations of intermediates of sphingolipid metabolism, such as palmitoyl dihydrosphingomyelin and behenoyl subgroups of shingomeylins, and a variety of sphingomyelin isomers. Regarding phospholipid metabolism, the HFD+TT800 group had (i) lower levels of trimethylamine N-oxide (TMAO), an intermediate of phospholipid metabolism (*p ≤* 0.05); and (ii) higher levels of glycerophosphorylcholine (GPC) and glycerophosphoinositol (GPI) (0.05 < *p <* 0.1).

TT supplementation resulted in higher levels of phosphatidylcholine intermediates (e.g., 1-palmitoyl-2-palmitoleoyl-GPC, 1-palmitoyl-2-oleoyl-GPC, and 1,2-dilinoleoyl-GPC, *p ≤* 0.05) and lysophospholipid intermediates (e.g., 1-lignoceroyl-GPC and 1-stearoyl-GPE, *p ≤* 0.05). TT supplementation also led to higher ratios for the levels of 1-(1-enyl-palmitoyl)-2-palmitoyl-GPC and 1-(1-enyl-palmitoyl)-2-linoleoyl-GPC (plasmalogen metabolites, 0.05 < *p <* 0.1), 1-dihmo-linolenyglycerol (a monoacylglycerol metabolite, *p ≤* 0.05), and glycosyl ceramide (a ceramides metabolite, 0.05 < *p <* 0.1). TT supplementation led to lower levels of palmitoyl-linoleoyl-glycerol (a metabolite of diacylglycerol, 0.05 < *p <* 0.1), mevalonate (an intermediate of mevalonate metabolism, *p ≤* 0.05), and beta-muricholate (a metabolite of bile acid metabolism, 0.05 < *p <* 0.1).

### 3.3. Effects of TT on Amino Acid Metabolism

Table 4 shows the effects of TT supplementation on serum amino acid metabolites in obese mice, again presented as ratios of (HFD+TT800)/HFD. Relative to the control HFD group, the HFD+TT800 group had higher levels of intermediates involved in amino acid metabolism, including serine (serine metabolism), alanine (alanine metabolism), pyroglutamine (glutamate metabolism), histidine and 1-methylhistamine (histidine metabolism), phenylpyruvate (phenylalanine metabolism), tyrosine and 2-hydroxylphenylacetate (tyrosine metabolism), serotonin and indoleacetate (tryptophan metabolism), 3-methylglutaconate and 3-methyl-2-oxobutyrate (leucine, isoleucine, and valine metabolism), S-methylcysteine (cysteine metabolism), 2-monomethylarginine (arginine metabolism), and creatine phosphate (creatine metabolism). The increases in two gluconeogenic amino acids, serine and alanine, could mean greater gluconeogenesis and improved glucose homeostasis to reduce obesity found in obese mice [15].

Furthermore, relative to the HFD group, the HFD+TT800 group had lower levels of intermediates of amino acid metabolism (Table 4), including N-acetylglycine (glycine metabolism), N-acetyl-3-methylhistidine and imidazole propionate (histidine metabolism), N2-acetyllysine, N2,N6-diacetyllysine, N6,N6,N6-trimethyllysine and 2-aminoadipate (lysine metabolism), kynurenate and xanthurenate (tryptophan metabolism), alpha-hydroxyisovalerate and ethylmalonate (leucine, isoleucine, and valine metabolism), N-acetyltaurine (taurine metabolism), 2-oxoarginine (arginine metabolism), 4-guanidinobutanoate (guanidine metabolism), and 2-hydroxybutyrate/2-hydroxyisobutyrate (glutathione metabolism).

### 3.4. Effects of TT on Carbohydrate Metabolism and Energy Cycle

**Figure 2:**
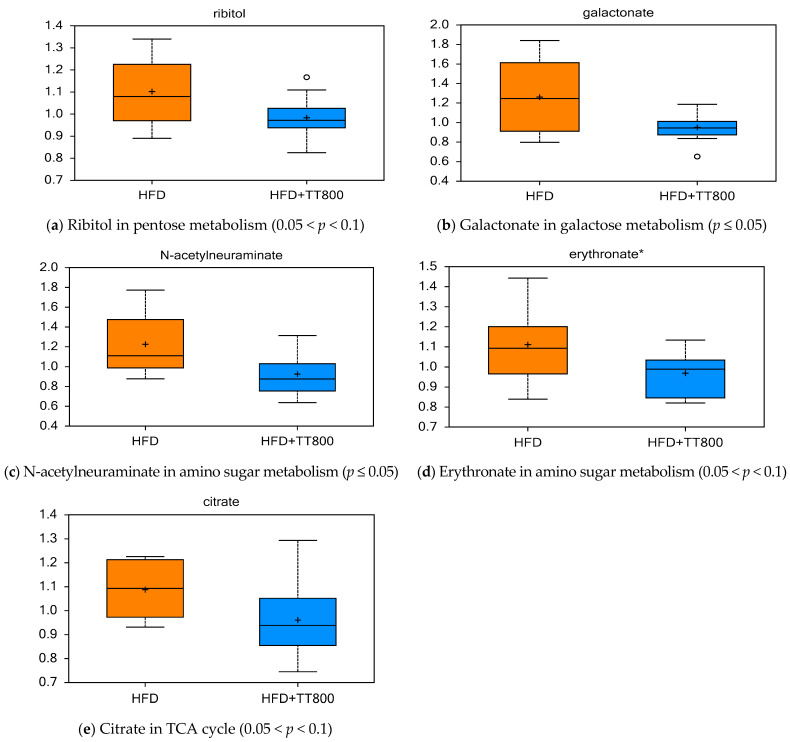
Effect of TT supplementation on carbohydrate metabolism. Importance of compounds relative to flux through carbohydrate metabolism and biosynthetic pathways in mice given TT. A lower level of citrate (**e**) in mice given TT might indicate a higher flux of TCA to support better energy production and use, and lower macronutrient carbon to support fat deposition when a HFD is fed. Generally, figures (**a**–**d**) suggest lower formation of these anabolic pathway intermediates. Each dietary group was comprised of *n* = 10 mice.

Figure 2 shows the effects of TT supplementation on carbohydrate metabolism and TCA cycle in obese mice. Compared to the control HFD group, the HFD+TT800 group had lower levels of serum ribitol (pentose metabolism, Figure 2a), galactonate (galactose metabolism, Figure 2b), N-acetylneuraminate (amino sugar metabolism, Figure 2c), erythronate (amino sugar metabolism, Figure 2d), and citrate (TCA cycle, Figure 2e).

### 3.5. Effect of TT on Cofactors and Vitamin Metabolism

**Figure 3:**
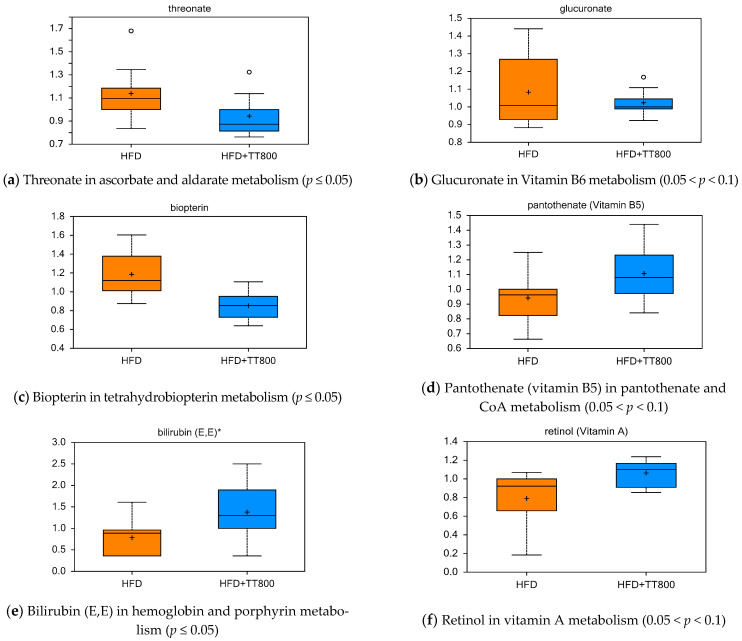

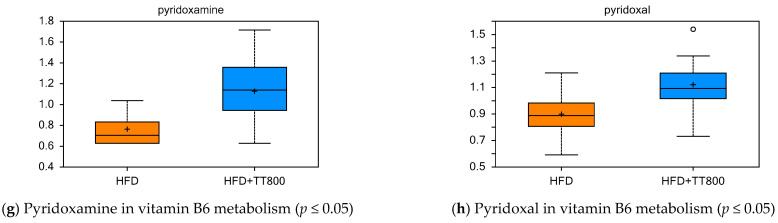
Effect of TT supplementation on serum cofactor and vitamin metabolites. TT may conserve pantothenate and CoA use for oxidative and biosynthetic reactions in intermediary metabolism, and intermediates of retinoic acid metabolism in mice, in contrast to a HFD that leads to anabolic responses for fat deposition. TT supplementation appears to reduce levels of biopterin for amino acid metabolism in support of neurotransmitter formation. Each dietary group was comprised of *n* = 10 mice.

The HFD+TT800 group had lower serum concentrations of threonate (ascorbate and aldarate metabolism, Figure 3a), glucuronate (vitamin B6 metabolism, Figure 3b), and biopterin (tetrahydrobiopterin metabolism, Figure 3c) than the HFD group (Figure 3). Furthermore, the HFD+TT800 group had higher concentrations of pantothenate (pantothenate and CoA metabolism, Figure 3d), bilirubin (hemoglobin and porphyrin metabolism, Figure 3e), retinol (vitamin A metabolism, Figure 3f), pyridoxamine (vitamin B6 metabolism, Figure 3g), and pyridoxal (vitamin B6 metabolism, Figure 3h).

### 3.6. Effect of TT on Nucleotide Metabolism

Table 5 shows the HFD+TT800 group had lower levels of intermediates involved in purine metabolism including (i) xanthine/inosine-containing metabolites, such as hypoxanthine, xanthine, xanthosine, 2′-deoxyinosine and allantoin; and (ii) guanine-containing metabolites, such as 7-methylguanine. In addition, the HFD+TT800 group had higher levels of intermediates involved in purine metabolism including (i) adenine-containing metabolites, such as adenosine-3′,5′-cyclic monophosphate, (ii) orotate-containing metabolites, such as dihydroorotate, and (iii) uracil-containing metabolites, such as uracil.

### 3.7. Effect of TT on Xenobiotics Metabolism

**Figure 4:**
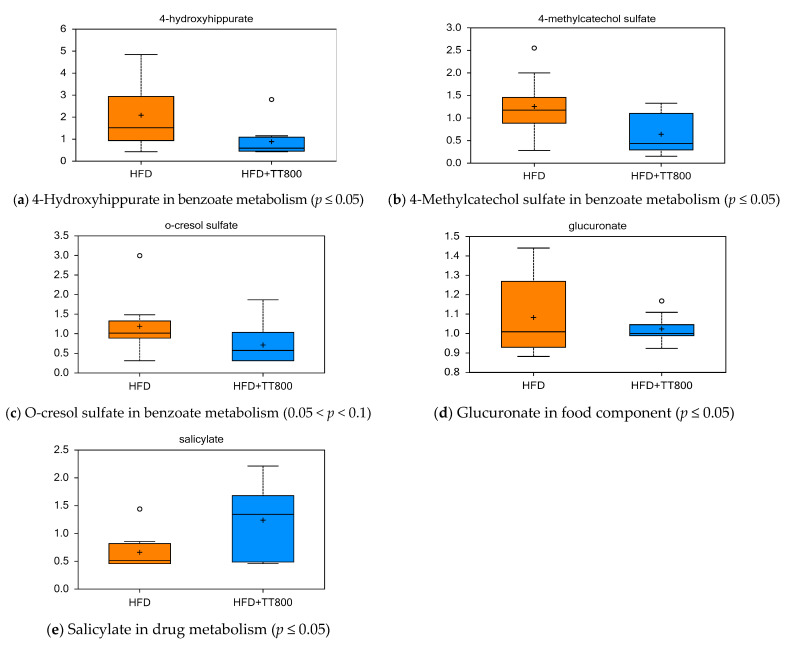
Effect of TT supplementation on serum xenobiotic metabolites. Supplementation with TT800 in mice fed a HFD may help reduce bacterial products of phenylalanine metabolism, and 4 hyrdoxyhippurate, as well as lower glucuranate. These compounds may be reduced with TT supplementation by changing gut microflora in mice. Each dietary group was comprised of *n* = 10 mice.

Figure 4 illustrates that TT-supplementation decreased the levels of intermediates involved in (i) benzoate metabolism (e.g., 4-hydroxyhippurate, Figure 4a, *p ≤* 0.05; 4-methylcartechol sulfate, Figure 4b, *p ≤* 0.05; and o-cresol sulfate Figure 4c, 0.05 < *p <* 0.1); and (ii) food component metabolism (glucoronate, Figure 4d, *p ≤* 0.05). TT supplementation may have reduced the amount of these compounds by changing gut microflora in mice. Annatto seed is a medium to high source of salicylates, consistent with the higher level of salicylates found in sera of mice given TT800 as shown in panel Figure 4e.

## 4. Discussion

The present study showed that 14 weeks of annatto-TT (90% δ-TT+10% γ-TT) supplementation significantly increased TT levels and reduced the intermediate metabolites of lipid peroxidation, dicarboxylic fatty acids (such as azelate, sebacate, 1,11-undecanedicarboxylate, dodecanedioate, tetradecanedioate and octadecanedioate) in mice fed a high fat diet. Dicarboxylic fatty acids are produced by fatty acid omega-oxidation, which can serve as a ’rescue pathway’ when beta-oxidation is impaired or overwhelmed (e.g., at times of high energy demand) [23]. The decrease in dicarboxylates in the HFD+TT800 group may suggest reduced omega-oxidation in response to TT supplementation, perhaps reflecting a more efficient β-oxidation of fatty acids or an enhanced ability to handle the excess load of fatty acids imposed by HFD. Fang et al. reported TT-enriched palm oil enhanced the interaction between the purified ligand-binding domain of PPARα with the receptor-interacting motif of coactivator PPARγ coactivator-1α in a cell-free in vitro system [24]. We previously reported PPARα mRNA was significantly higher in animals fed with an HFD supplemented with TT400 in the diet, relative to those fed without TT400 supplementation [8]. Thus, in the present study, the observed decrease in intermediate metabolites of dicarboxylic fatty acids in the HFD+TT800 group confirms one of the molecular mechanisms for the anti-oxidation activity of TT. Specifically, in mice given TT compared to the control group, medium chain fatty acids and fatty acid metabolism (acyl glycine) decreased, however, carnitine metabolism and fatty acid acyl carnitine (medium and long-chain fatty acids) increased.

Some oxylipins were changed favorably in mice supplemented with TT, and the ratio values of HFD+TT800/HFD for 13-HODE and 9-HODE, 14 HDoHe/17-HDoHe, and thromboxane B2 were lower, suggesting actions of lower acute pain and inflammation and auto-oxidation of DHA. The amount for 14 HDoHe/17-HDoHe showed a lower ratio which may support lower circulating triglycerides as we previously reported in male mice given TT with a high fat diet compared mice not given TT [8]. Furthermore, the lower value for 12-HETE would suggest a reduced production of growth factor cytokines. The reduced arachidonic acid COX metabolite 12 HHTrE would lower inflammation in obese mice.

Sphingomyelins are a class of phospholipids that are found in animal cell membranes and form lipid microdomains with cholesterol [25]. The unique composition of sphingomyelins allows them to play important roles in both membrane stabilization and lipid signaling [25]. In addition to the role of sphingolipids as structural components of cell membranes, sphingolipids also function as intracellular and extracellular mediators that regulate cellular processes including cell survival, proliferation, apoptosis, differentiation, migration, and immune processes [26]. Emerging evidence suggests that biosynthesis or metabolism of sphingolipids is altered in obesity, diabetes, and cardiovascular diseases [25,26,27]. Our study demonstrated a substantial accumulation of several sphingomyelin components in the HFD+TT800 group. The increase in the sphingomyelin pool observed in the HFD+TT800 group may correlate with several factors including fatty acid availability, ceramide utilization, membrane turnover, and/or uptake. Evidence suggests that TT supplementation leads to intracellular accumulation of dihydroceramide and sphinganine, two key sphingolipid intermediates in the de novo synthesis of the sphingolipid pathway that contribute to TT-supplemented apoptosis of adipocytes [28]. In the present study, an increase in glycosyl ceramide and sphingomyelin intermediates by TT supplementation could indicate changes in the de novo synthesis of sphingolipids, and a role in the pro-death effect on adipocytes in prevention of obesity [28].

Mice given TT compared to the controls in our study showed higher levels of PC, an abundant phospholipid of mammalian cells and subcellular organelles. There was a higher amount of complex lipid composition supporting phospholipid metabolism (e.g., GPC, GPI), PC (e.g., 1-palmitoyl-2-oleoyl-GPC, 1-2-dilinoleoyl-GPC) and lysophospholipid (e.g., 1-lignoceroyl-GPC, 1-stearoyl-GPE) in the TT-supplemented obese mice compared to the controls. These differences likely suggest that TT could play a supporting role in regulating lipid, lipoprotein, and energy metabolism.

In our current study, TT-supplemented obese mice had significantly lower trimethylamine N-oxide (TMAO) in serum relative to the controls. TMAO has been shown in animal models to up-regulate macrophage receptors associated with atherosclerosis [29]. Elevated serum concentrations of TMAO have been linked to adverse cardiovascular outcomes in humans [29,30] and TMAO has been identified as a cardiovascular disease risk factor and biomarker in humans [31]. TMAO is also a product of phospholipid and carnitine metabolism by the intestinal microbiome. Li et al. reported the serum content of TMAO and inflammatory cytokines (i.e., interleukin-1, interleukin-2, and monocyte chemoattractantprotein-1) were significantly higher in the T2DM rats than those in the control [32]. Authors also reported that the gut microbiota of T2DM rats had decreased abundance of beneficial bacteria, such as *Allobaculum*, *Bifidobacterium*, *Eubacterium*, and *Anaerotruncus*, while those of opportunistic pathogens (e.g., *Enterococcus*, *Corynebacterium*, *Aerococcus*, and *Facklamia*) were increased, suggesting the role of TMAO in modification of gut microbiome during the development of T2DM [32]. In our previous study [15], we reported that dietary TT supplementation increased Bacteroidetes/Firmicutes ratio and the number of bacteria which belong to Clostridiales order, especially the Oscillospira genus (Firmicutes phylum) was decreased by two-fold in the feces of obese mice. The current finding that TT supplementation favors a beneficial gut microbiome [15] along with decreased serum TMAO in obese mice suggests a potential for TT to improve glucose homeostasis, which is consistent with our previous observation that TT supplementation improved glucose and insulin tolerance in HFD mice [8,9].

Dietary supplementation with TMAO decreased the expression of a number of bile acid transport genes in the liver (Cyp7a1 and Cyp27a1) adversely affecting cholesterol elimination [30] and blocking reverse cholesterol transport, providing another potential link between TMAO and vascular disease. Further, we previously reported that TT supplementation lowers triglyceride accumulation and inflammation in liver of obese mice [8]. Thus, the decreased serum TMAO level in the TT-treated obese mice compared to controls provides a supportive mechanism for the role of TT in promoting metabolically healthy obesity.

Lysophosphatidylcholine (LPC) is an important signaling molecule with diverse biological functions [33]. LPC is involved in inflammation [34] and insulin resistance in obesity [35] and can exert positive effects on glucose metabolism [36]. Barber et al. (2012) has demonstrated that exposing mice to chronic HFD markedly reduced plasma LPC (14:0, 15:0, 20:0, 20:1, 20:5, 16:1, and 18:1) along with an increase in fat mass and the development of glucose intolerance and hyperinsulinemia [37]. Such a reduction in LPC species observed in obese mice was also reported in plasma from obese animals [37] and obese T2DM individuals [38]. Yea et al. (2009) demonstrated that LPC activates adipocyte glucose uptake and lowers blood glucose levels in murine models of diabetes [36]. Our findings that TT increases LPC suggested reduced inflammation.

One of the earliest observed biological activities of TT is the ability to downregulate the mevalonate pathway via suppression of its rate-limiting enzyme, 3-hydroxy-3-methylglutaryl coenzyme A reductase (HMGCR) [38]. Among the TT isomers, delta- and gamma-TTs have been shown to be the most potent in suppressing HMGCR [39]. The TT appears to downregulate HMGCR at the transcriptional level by mimicking the action of its transcription factors or enhance the ubiquitination and degradation of HMGCR at the post-translational level [40]. The reduced serum level of mevalonate in TT-supplemented mice compared to controls is consistent with these previous findings.

The association of obesity, insulin resistance, and chronic oxidative stress/low-grade inflammation has been evident for more than a decade [41]. The pathways for ROS production and oxidative stress are upregulated in a coordinated way in HFD mice, along with hyperglycemia and insulin resistance [42]. Insulin metabolism has been linked to oxidized LDL [42]. For instance, Linna et al. has shown the highest HOMA-IR quartile consistently had the highest concentrations of oxidized-LDL and ratio of oxidized-LDL/HDL-cholesterol [42], suggesting a strong positive association between insulin resistance and damage caused by oxidized lipids in T2DM individuals. Bioactive oxidized linoleic acid metabolites, such as 9- and 13-hydroxyoctadecadienoic acid (9-HODE+13-HODE), have been linked to oxidative stress, inflammation and numerous pathological and physiological states [43]. In the present study, TT supplementation decreased the levels of 9-HODE and 13-HODE in serum of obese mice, indicating TT mitigated the potential of linoleic acid metabolites to oxidized products.

**Figure 5:**
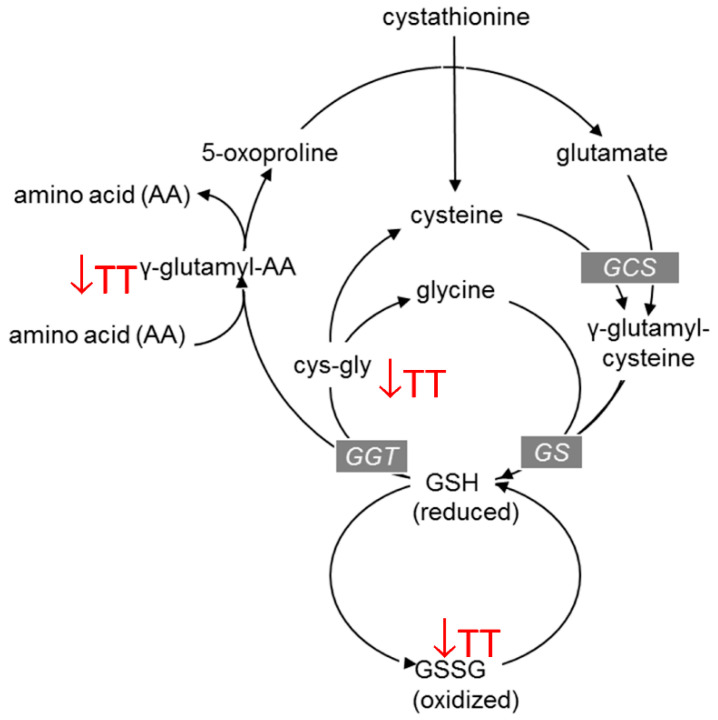
Effects of TT supplementation on the methionine-cysteine cycle and GSH/GSSH redox status. Supplementation of mice fed a HFD with TT alters the amounts of intermediates in amino acid metabolism and oxidized glutathione that reflect a lowered ratio of reduced glutathione/oxidized glutathione (GSH/GSSH), and thus, a lowered oxidative stress. TT, tocotrienol.

We previously reported TT supplementation improved glucose homeostasis (improved insulin resistance and decreased hyperglycemia) in obese mice via a reduction of inflammation and oxidative stress [8,15]. We showed that compared to the HFD group without TT, TT supplementation to an HFD lowered values of TNF-α, IL-6 and MCP-1 in adipose tissue [8,15]. Fang et al. also demonstrated the tocotrienol-rich fraction of palm oil improved whole body glucose utilization and insulin sensitivity of diabetic Db/Db mice by selectively regulating PPAR target genes [24] In the current study, the HFD +TT800 mice showed a relative decrease in some of the oxidative stress and stress response markers. As shown in Figure 5, the methionine/cysteine circuit is tightly linked to glutathione production to actively manage oxidative stress. Noticeable (*p* > 0.1) decreases in the ratios for oxidized glutathione (0.4056), cysteine-glutathione disulfide (0.7438), methionine sulfoxide (0.974), 5-oxoproline (0.949), and cystine (0.8775) were observed in TT-treated mice in relation to control mice. Reduction in these metabolites is reminiscent of an improved redox environment and might be an indication of lower oxidative insult by TT supplementation. In addition, lower activity of the γ-glutamyl cycle, which is important for recycling and regeneration of GSH, was noted in TT-treated mice, as evidenced by a decline in γ-glutamyl amino acids. Furthermore, compared to the HFD mice, the oxidative stress markers including oxidized lipids (12,13-DiHOME, 9-HODE + 13-HODE) and inflammatory molecule kynurenate were significantly reduced while an antioxidant marker threonate was significantly increased in the HFD +TT800 mice. Thus, the observed decrease in the levels of these oxidative stress markers provides added support for the notion that treatment with TT lowers HFD-induced oxidative stress.

Xanthine oxidase (XO) oxidizes hypoxanthine to xanthine and uric acid, producing hydrogen peroxide. A large body of work has demonstrated that enhanced XO activity is considered a damage signal, contributing to a damage-associated molecular pattern [44]. Endothelial xanthine oxidoreductase (XOR) together with NADPH oxidase and nitric oxide (NO) synthase plays a physiological role in inflammatory signaling, the regulation of NO production and vascular function [44]. The role of XOR in adipogenesis is also connected with insulin resistance and obesity, two main features of T2DM [44]. In the present study, consistent with TT-mediated reduction in oxidative stress, we observed a significant decrease in purine breakdown products such as hypoxanthine, xanthine, xanthosine and allantoin in obese mice, suggesting TT reduced XO activity and ROS production and protected against HFD-induced oxidative stress. Collectively, these results point towards TT as a potent antioxidant against obesity-induced oxidative damage.

A noticeable increase in vitamin B metabolites including pyridoxamine and pyridoxal was displayed by mice supplemented with TT. Vitamin B6 is an essential cofactor in various transamination, decarboxylation, glycogen hydrolysis, and synthesis pathways involving carbohydrate, sphingolipid, amino acid, heme, and neurotransmitter metabolism. Since the gut microbiota is associated with the biosynthesis of B vitamins, the observed fluctuation in vitamin B molecules in the HFD+TT800 animals could be linked to TT-mediated favorable alterations in microbiome composition [15]. A consequential increase in serotonin (a tryptophan-derived neurotransmitter that has roles in mediating behavior, mood, gut movement, growth, and learning) was noted in the TT-supplemented obese mice. Consistent with this finding, we also observed changes in the benzoate pathway intermediates (4-hydroxyhippurate and 4-methylcatechol sulfate), which are derived from microbiome metabolism of aromatic amino acids. Collectively, these changes are reflective of favorable gut microbial influences associated with TT supplementation [15].

## 5. Conclusions

This global metabolomic profiling study was conducted to gain an understanding of the changes in metabolites associated with TT supplementation in HFD-induced obesity in mice. Overall, the results from the serum profiling demonstrate alterations in biochemical pathways, including those of amino acids, lipid oxidation and peroxidation, oxidative stress, sphingolipids, and xanthine oxidase along with changes in serum metabolites that may be related to the intestinal microbiome. As a path forward, it is important to measure metabolic changes under TT treatment using a complex lipid platform which will shed light on the comprehensive changes in lipid metabolism with TT supplementation. Additionally, research on adipose tissue adipokines and composition of fatty acids in obesity should reveal the role of TT in inflammation and fatty acid metabolism in regard to oxylipins and endocannabinoids. Finally, the level of TT used is well within the safety range of TT [45], suggesting the potential of TT for clinical applications. It is not only the potent antioxidant capacity, but also the safety and efficacy of TT that enhances their clinical potential [45].

## Figures and Tables

**Table 1:** Ingredient composition of the experimental diets (g/kg).

Ingredient	HFD	HFD+TT800
Casein, 80 Mesh	267.1	267.1
L-Cystine	4	4
Maltodextrin 10	166.9	166.9
Sucrose	91.9	91.9
Cellulose, BW 200	66.8	66.8
Soybean oil ^a^	33.38	33.38
Lard	327.2	327.2
Mineral mix ^b^, S10026B	13.4	13.4
Dicalcium phosphate	17.4	17.4
Calcium carbonate	7.3	7.3
Potassium citrate, 1 H_2_O	22	22
Vitamin mix ^c^, V13401 (without E)	13.4	13.4
Vitamin E acetate (500 IU/gm)	0.1	0.1
Choline bitartrate	2.7	2.7
Tocotrienol ^d^	0	0.56

HFD, high-fat diet control group; HFD+TT800, a high-fat diet supplemented with tocotrienol (TT800) at 800 mg/kg diet. ^a^ Soybean oil tocopherol stripped (catalog number: 404365, Dyets Inc., Bethlehem, PA, USA). ^b^ Mineral mix provides (g/kg diet): calcium phosphate, dibasic, 260; calcium carbonate, 110; potassium citrate,1H_2_O, 330; sodium chloride, 51.8; magnesium oxide, 8.38; magnesium sulfate, 7H_2_O, 51.52; chromium K sulfate, 12H_2_O, 0.385; cupric carbonate, 0.21; sodium fluoride, 0.04; potassium iodate, 0.007; ferric citrate, 4.2; manganous carbonate, 2.45; ammonium molybdate, 4H_2_O, 0.06, sodium selenite, 0.007, zinc carbonate, 1.12; sucrose, 179.821. ^c^ Vitamin mix provides (g/kg diet): vitamin A acetate (500,000 IU/g), 0.8; vitamin D3 (100,000 IU/g), 1.0; vitamin K1 (menadione sodium bisulfite, 62.5% menadione), 0.08; biotin (1%), 2.0; cyanocobalamin (B12) (0.1%), 1.0; folic acid, 0.2; nicotinic acid, 3.0; calcium pantothenate, 1.6; pyridoxine-HCl, 0.7; riboflavin, 0.6; thiamine HCl, 0.6; sucrose, 988.42. ^d^ Tocotrienols was an extract of annatto oil containing 86.1% δ-tocotrienol and 13.9% γ-tocotrienol (American River Nutrition, Hadley, MA, USA). High-performance liquid chromatography determined the purity content to be 71.3%.

**Table 2:** Effects of TT supplementation on serum fatty acid metabolites in mice fed HFD compared to the HFD control group.

Sub Pathway	Metabolite Name	HFD+TT800/HFD
Fatty acid branched	13-methylmyristate	**0.80**
Medium chain fatty acid	heptanoate (C7:0)	**1.13**
	pelargonate (C9:0)	**0.84**
	10-undecenoate (C11:1n1)	**0.69**
Fatty acid monohydroxy	2-hydroxyoctanoate	**0.84**
	9-HODE + 13-HODE	**0.71**
	14-HDoHE/17-HDoHE	**0.82**
Fatty acid, dicarboxylate	2-hydroxyadipate	**0.69**
	suberate (octanedioate)	**0.81**
	azelate (nonanedioate)	**0.79**
	sebacate (decanedioate)	**0.77**
	Undecanedioate	**0.81**
	1,11-undecanedicarboxylate	**0.75**
	dodecanedioate	**0.81**
	tetradecanedioate	**0.80**
	hexadecanedioate	**0.85**
	Octadecanedioate	**0.79**
Eicosanoid	Thromboxane B2	**0.45**
	12-HHTE	**0.72**
	12-HHTrE	**0.47**
Endocannabinoid	oleoyl ethanolamide	**1.07**
	palmitoyl ethanolamide	**1.09**
	linoleoyl ethanolamide	**1.11**
Fatty acid acyl glycine	Valerylglycine	**0.61**
	Hexanoylglycine	**0.51**
	heptanoyl glycine	**0.53**
	3,4-methylene heptanoylglycine	**0.63**
	N-octanoylglycine	**0.57**
	N-palmitoylglycine	**0.93**
Fatty acid acyl carnitine	hexanoylcarnitine (C6)	**0.71**
	decanoylcarnitine (C10)	**1.29**
	lignoceroylcarnitine (C24)	**1.18**
Carnitine	Carnitine	**1.22**

Metabolite values are expressed as the ratio of HFD+TT800/HFD which is the fold change of the treated HFD+TT800 group compared to the HFD control group. A ratio greater than 1 indicates a value larger for the treated group (HFD+TT800) and less than 1 the value for HFD+TT800 group is lower compared to the HFD control group. Green indicates fold reduction and red is for fold increase by TT supplementation with *p* ≤ 0.05 obtained from the Student’s *t*-test. Yellow indicates fold reduction and pink is for fold increase by TT supplementation with 0.05 < *p <* 0.1 that was obtained from the Student’s *t*-test.

**Table 3:** Effects of TT supplementation on serum sphingolipid, phospholipid, and other lipid metabolites in mice fed HFD compared to the HFD control group.

Sub Pathway	Metabolite Name	HFD+TT800/HFD
Sphingolipid Metabolism	palmitoyl dihydrosphingomyelin (d18:0/16:0)	1.08
	palmitoyl sphingomyelin (d18:1/16:0)	0.31
	behenoyl sphingomyelin (d18:1/22:0)	1.32
	sphingomyelin (d17:1/16:0, d18:1/15:0, d16:1/17:0)	1.19
	sphingomyelin (d18:2/16:0, d18:1/16:1)	1.11
	sphingomyelin (d18:1/18:1, d18:2/18:0)	1.26
	sphingomyelin (d18:1/20:1, d18:2/20:0)	1.24
	sphingomyelin (d18:1/24:1, d18:2/24:0)	1.14
	sphingomyelin (d18:2/21:0, d16:2/23:0)	0.78
	sphingomyelin (d18:2/24:2)	1.21
	sphingomyelin (d18:1/22:2, d18:2/22:1, d16:1/24:2)	1.21
	sphingomyelin (d18:2/18:1)	1.24
	sphingomyelin (d18:1/19:0, d19:1/18:0)	0.72
Phospholipid Metabolism	glycerophosphorylcholine (GPC)	1.25
	glycerophosphoinositol (GPI)	1.20
	trimethylamine N-oxide (TMAO)	0.58
Phosphatidylcholine (PC)	1-palmitoyl-2-palmitoleoyl-GPC (16:0/16:1)	1.15
	1-palmitoyl-2-oleoyl-GPC (16:0/18:1)	1.12
	1,2-dilinoleoyl-GPC (18:2/18:2)	1.24
Lysophospholipid	1-lignoceroyl-GPC (24:0)	1.39
	1-stearoyl-GPE (18:0)	1.17
Plasmalogen	1-(1-enyl-palmitoyl)-2-palmitoyl-GPC (P-16:0/16:0)	1.23
	1-(1-enyl-palmitoyl)-2-linoleoyl-GPC (P-16:0/18:2)	1.17
Monoacylglycerol	1-dihomo-linolenylglycerol (20:3)	1.66
Diacylglycerol	palmitoyl-linoleoyl-glycerol (16:0/18:2)	0.40
Ceramides	glycosyl ceramide (d18:1/20:0, d16:1/22:0)	1.21
Mevalonate Metabolism	Mevalonate	0.84
Primary Bile Acid Metabolism	beta-muricholate	0.69

Metabolite values are expressed as the ratio of HFD+TT800/HFD which is the fold change of the treated HFD+TT800 group compared to the HFD control group. A ratio greater than 1 indicates a value larger for the treated group (HFD+TT800) and less than 1 the value for HFD+TT800 group is lower compared to the HFD control group. Green indicates fold reduction and red is for fold increase by TT supplementation with *p* ≤ 0.05 obtained from the Student’s *t*-test. Yellow indicates fold reduction and pink is for fold increase by TT supplementation with 0.05 < *p* < 0.1 that was obtained from the Student’s *t*-test.

**Table 4:** Effects of TT supplementation on serum amino acid metabolites in mice fed HFD compared to the HFD control group.

Sub Pathway	Biochemical Name	HFD+TT800/HFD
Glycine, Serine, and Threonine Metabolism	N-acetylglycine	0.75
	Serine	1.13
Alanine and Aspartate Metabolism	Alanine	1.17
Glutamate Metabolism	Pyroglutamine	1.15
Histidine Metabolism	Histidine	1.06
	N-acetyl-3-methylhistidine	0.68
	Imidazole propionate	0.35
	1-Methylhistamine	1.28
Lysine Metabolism	N2-acetyllysine	0.76
	N2,N6-diacetyllysine	0.83
	N6,N6,N6-trimethyllysine	0.76
	2-Aminoadipate	0.84
Phenylalanine Metabolism	Phenylpyruvate	1.28
Tyrosine Metabolism	Tyrosine	1.12
	2-Hydroxyphenylacetate	1.29
Tryptophan Metabolism	Kynurenate	0.70
	Xanthurenate	0.43
	Serotonin	1.39
	Indoleacetate	1.31
Leucine, Isoleucine, and Valine Metabolism	3-Methylglutaconate	2.00
	Alpha-hydroxyisovalerate	0.88
	Ethylmalonate	0.84
	3-Methyl-2-oxobutyrate	1.35
Methionine, Cysteine, SAM, and Taurine Metabolism	S-methylcysteine	1.42
	N-acetyltaurine	0.77
Urea cycle, Arginine, and Proline Metabolism	2-Oxoarginine	0.81
	N-monomethylarginine	1.22
Creatine Metabolism	Creatine phosphate	1.86
Guanidino and Acetamido Metabolism	4-Guanidinobutanoate	0.80
Glutathione Metabolism	2-Hydroxybutyrate/2-Hydroxyisobutyrate	0.75

Metabolite values are expressed as the ratio of HFD+TT800/HFD which is the fold change of the treated HFD+TT800 group compared to the HFD control group. A ratio greater than 1 indicates a value larger for the treated group (HFD+TT800) and less than 1 the value for HFD+TT800 group is lower compared to the HFD control group. Green indicates fold reduction and red is for fold increase by TT supplementation with *p* ≤ 0.05 that was obtained from the Student’s *t*-test. Yellow indicates fold reduction and pink is for fold increase by TT supplementation with 0.05 < *p*< 0.1 that was obtained from the Student’s *t*-test.

**Table 5:** Effects of TT supplementation on serum nucleotide metabolites in mice fed HFD compared to the HFD control group.

Sub Pathway	Biochemical Name	HFD+TT800/HFD
Purine metabolism, xanthine/Inosine containing	Hypoxanthine	0.23
	Xanthine	0.41
	Xanthosine	0.53
	2′-Deoxyinosine	0.15
	Allantoin	0.85
Purine metabolism, Adenine containing	Adenosine-3′,5′-cyclic monophosphate (cAMP)	1.55
Purine metabolism, Guanine containing	7-Methylguanine	0.74
Purine Metabolism, Orotate containing	Dihydroorotate	1.55
Pyrimidine Metabolism, Uracil containing	Uridine	1.18

Metabolite values are expressed as the ratio of HFD+TT800/HFD which is the fold change of the treated HFD+TT800 group compared to the HFD control group. A ratio greater than 1 indicates a value larger for the treated group (HFD+TT800) and less than 1 the value for HFD+TT800 group is lower compared to the HFD control group. Green indicates fold reduction and red is for fold increase by TT supplementation with *p* ≤ 0.05 that was obtained from the Student’s *t*-test. Yellow indicates fold reduction and pink is for fold increase by TT supplementation with 0.05 < *p* < 0.1 that was obtained from the Student’s *t*-test.

## Data Availability

Available upon request.
